# Deciphering immunosuppressive niches by spatial single-cell proteomics: spatial interaction networks and translational opportunities

**DOI:** 10.3389/fimmu.2026.1904684

**Published:** 2026-07-29

**Authors:** Hao Chai, Lang Wu, Xiaopeng Chen, Zhiming Ma, Chengwei Yang, Minghao Li

**Affiliations:** People's Hospital of Ningxia Hui Autonomous Region, Third Clinical Medical College, Ningxia Medical University, Yinchuan, China

**Keywords:** CODEX, immunosuppressive niche, precision immunotherapy, spatial interaction network, spatial single-cell proteomics, tumor microenvironment

## Abstract

Although immunocheckpoint blocking therapy has been successful in a variety of cancers, its long-term response is still rare. The difference between initial clinical benefits and persistent disease control reflects the complexity of intratumor immunomodulation. People’s attention is increasingly shifting from a single group of cells to the spatial environment in which these cells live. Tumor cells, immune cells and interstitial components cannot function independently; on the contrary, they form a local tissue structure, and their composition and structure affect immune activity. Technologies such as Co-Detection by Indexing (CODEX), Imaging Mass Cytometry (IMC), and multiple ion beam imaging (MIBI) can now directly detect these spatial relationships while measuring dozens of proteins at a single cell resolution. Therefore, the spatially defined immunosuppressive niche has become an important framework for explaining how local cell interactions lead to immune dysfunction and therapeutic resistance. These studies reveal that immune cells, stromal cells, and malignant cells form a highly ordered spatial network, synergistically regulated through direct contact, cytokine signaling, and metabolic interactions. This coordinated cellular network forms a local immunosuppressive microenvironment, thereby maintaining immune escape, tumor progression, and treatment resistance. Recent research has also uncovered previously unknown immunosuppressive cell states and their spatial interaction patterns, characteristics closely related to disease prognosis, recurrence risk, and immunotherapy response. Therefore, tumor immunology research is shifting from the characterization of single cell populations to the exploration of spatially organized functional ecosystems. This article reviews the latest advances in the field of space-based single-cell proteomics and summarizes current research findings on the cellular composition, spatial structure, and regulatory mechanisms of the immunosuppressive microenvironment. Furthermore, we explore the emerging clinical value of space biomarkers and interaction networks in patient stratification, therapeutic target identification, and precision immunotherapy.

## 1 Introduction

Immune checkpoint inhibitors (ICIs) have significantly improved the survival of patients with a variety of malignant tumors and promoted tumor therapy into the immune era (1). However, only a part of patients can achieve sustained response to immunotherapy, and primary or secondary drug resistance is still widespread, suggesting that the mechanism of tumor immune escape is far more complex than traditional understanding. The high heterogeneity of tumor mircroenvironment (TME) is one of the core bottlenecks limiting the efficacy of immunotherapy (2, 3). Previous studies mainly focused on the expression status of immune checkpoint molecules such as programmed death receptor 1 (PD-1), programmed death ligand 1 (PD-L1) and cytotoxic T lymphocyte-associated antigen 4 (CTLA-4), but the ability of these indicators to predict the prognosis and treatment response of patients still has obvious limitations (4). However, there is still a high degree of heterogeneity in treatment response between tumor types and individual patients. Increasing evidence has shown that tumor progression and treatment outcome are not only dependent on the number of immune cells in the TME, but also influenced by complex interactions among tumor cells, immune cells, and stromal cells (5, 6). Therefore, understanding the organization of the TME has become a central challenge in tumor immunology.

With the development of single-cell sequencing technology, researchers can analyze the cellular composition and molecular heterogeneity of tumor tissues at the single-cell level, thereby discovering a large number of new immune cell subsets and functional states, which has been widely used in the study of solid tumors such as hepatocellular carcinoma(HCC) (7). However, traditional single-cell transcriptome sequencing requires the dissociation of tissues, so the original spatial location information of cells is inevitably lost (8, 9). Spatial location is precisely the premise of functional interaction between cells, because at the level of tumor immune microenvironment (TIME) and metabolic reprogramming, the functional status of immune cells not only depends on their own molecular characteristics, but also is significantly affected by the composition of neighboring cells, local microenvironment and spatial tissue structure (10). Therefore, it is difficult to fully explain the immune regulatory mechanism in the TME only by relying on the information of cell composition. Although traditional pathology provides certain spatial information, it is limited by the subjectivity of human observation and the number of detectable markers (11). In recent years, the emerging spatial omics technology provides a new research paradigm for analyzing the structure of tumor tissues. Among them, CODEX, IMC, and MIBI can achieve the simultaneous detection of dozens or even hundreds of protein markers while preserving the integrity of tissue structure (12). These techniques can not only identify cell types and functional states, but also accurately characterize the proximity relationship between cells, spatial aggregation patterns and tissue structure characteristics (13). Studies based on spatial information have found that cells in TME are not randomly distributed, but form cellular communities and spatial neighborhoods with specific structures and functions (14); These local regions composed of specific cell populations that maintain an immunosuppressive state—characterized by coordinated cellular interactions and suppressive signaling within spatially confined microdomains—have been gradually defined as “immunosuppressive niche” (15, 16). Different from the traditional concept of “immune checkpoint”, the immunosuppressive niche emphasizes the regulatory role of the spatial interaction network—defined as a graph-based representation of cell–cell spatial proximity and functional interactions—between cells on immune function (5). Therefore, the research focus is gradually shifting from a single cell or molecule to a spatial functional unit composed of multiple cells. In 2024, Qiu et al. used CODEX technology to analyze the spatial protein profile of 401 HCC cases and found that vimentin(VIM)-highly macrophages co-existed with regulatory T cells (Tregs) in space and enhanced Treg inhibitory function through IL-1β. This spatial co-occurrence pattern is significantly correlated with immunotherapy response, and a prognostic classification based on spatial features has been established (17). At the same time, other spatial proteomics-based discoveries are continuously expanding the understanding of this field. Yang et al. used IMC technology to analyze spatial proteins in 92 surgical specimens and found that there were significant differences in spatial proteome characteristics between primary and recurrent HCC, and recurrent HCC showed a more immunosuppressive spatial ecosystem (18). Massoudi et al. systematically summarized that the spatial organization of HCC TME is a key factor determining progression, immune escape and therapeutic response, and spatial analysis provides a new basis for targeted therapy and risk stratification (19).

In recent years, the progress of space single-cell proteomics has fundamentally changed our understanding of tumor immunology, and achieved high-resolution and spatial resolution imaging of cell tissue in the tumor microenvironment. However, despite the rapid development of technology, most existing studies and reviews are still mainly at the descriptive level, either focusing on spatial analysis techniques or focusing on the bibliography of immunosuppressive cell components, and lacking a unified framework for interpretation. This paper proposes the “space immunosuppression ecosystem framework”, which integrates spatial proteomics technology, the immune microenvironment of space tissue, the directional intercellular communication network and the principle of pan-cancer convergence to build a coherent system-level structure. The framework shifts the conceptual focus from individual cell type to a spatially organized and functionally coordinated immune ecosystem, providing a unified perspective for explaining the heterogeneity of tumor immune escape, therapeutic drug resistance and reaction between different cancer types. We believe that this system-level spatial perspective is crucial to the development of the next generation of precision immunotherapy and patient stratification strategies based on spatial information.

## 2 Spatial single-cell proteomics promotes immune niche research

### 2.1 From cellular composition analysis to spatial tissue organization

Over the past decade, single-cell RNA sequencing (scRNA-seq) has substantially advanced our understanding of the TME (20). This technology has enabled the identification of numerous previously unrecognized immune cell subsets, including exhausted T cells, TREM2+ macrophages, SPP1+ macrophages, and Tregs with distinct functional states (21–23). However, tissue dissociation into single-cell suspensions inevitably results in the irreversible loss of spatial information (24). Consequently, the physical proximity between cells, tissue structure, and local microenvironment characteristics could not be preserved. This deficiency is particularly prominent in the study of tumor immunity. Many immune regulatory processes have obvious distance dependence (25). For example, cytokine diffusion is limited, and receptor-ligand interactions often require direct cell contact or short-distance communication. Thus, cells with the same transcriptional state may serve vastly different functions in different tissue regions. Early pathological studies have found that the spatial distribution pattern of tumor infiltrating lymphocytes (TILs) can better reflect the prognosis of patients than its absolute number (26). Fridman et al. proposed the concept of “Immunoscore” in colorectal cancer and found that the density of CD3+ and CD8+T cells in the tumor core area and invasive marginal area was significantly correlated with the survival of patients (27, 28). This finding suggests that spatial organization itself has independent biological significance. Therefore, the study of tumor immunity has gradually shifted from “cellular composition analysis” to “spatial organization analysis”.

### 2.2 Major spatial single-cell proteomics platforms and their characteristics

The rapid development of space single-cell proteomics has given rise to a variety of high-throughput imaging and sequencing platforms, each of which focuses on spatial resolution, molecular coverage and clinical applicability (29). Together, these techniques have achieved the preservation of tissue structure while drawing cell phenotypic maps *in situ*, thus providing unprecedented insights into the tissue structure of the tumor microenvironment (30–32). **Table 1** summarizes representative spatial proteomics and spatial transcriptomics platforms rather than providing an exhaustive list of all available technologies. Although the detection principles of these technologies are fundamentally different, covering from antibody-based imaging mass spectrometry flow cytometry to nucleic acid-based spatial transcriptomics and artificial intelligence-assisted virtual reconstruction methods, their common feature is that they can retain tissue structure *in situ*, so that they can be fixed in the natural microenvironment of cells. Analyze the spatial relationship between cells. The current proteomics platform can be roughly divided into three categories: imaging-based proteomic methods (such as IMC, CODEX and MIBI) (17, 43, 44), cyclic imaging methods based on high-throughput fluorescence (including CyCIF and MACSima) (45, 46) and spatial transcriptomics or sequencing-based spatial reconstruction systems (such as MERFISH, Xenium and Stereo-seq) (38, 47, 48). Imaging-based methods can provide high spatial fidelity at single-cell or subcellular resolutions, but their multiple analysis capabilities and throughput are relatively low. In contrast, the sequencing-based spatial transcriptomics platform can provide higher molecular depth, but there may be indirect problems such as reduced protein horizontal resolution and spatial protein expression inference. Artificial intelligence-driven virtual spatial proteomics is an emerging direction, which combines histopathological imaging with deep learning models to infer spatial protein distribution on a large scale (41, 49).

**Table 1:** Comparison of major spatial single-cell proteomics platform.

Category	Platform	Modality	Spatial resolution	Multiplex capacity	Biological layer captured	Tissue compatibility	Ecosystem modeling capability	Clinical readiness	Strengths	Key limitations	Representative references
Imaging-based spatial proteomics	CODEX/PhenoCycler	Antibody-based cyclic imaging	Single-cell	40–120+ proteins	Proteome (protein-level)	FFPE/fresh frozen	High (cell–cell interaction networks)	II–III (translational research)	High spatial fidelity; robust cell interaction mapping	Workflow complexity; computational burden	(33)
	IMC	Metal-tag antibody imaging	Single-cell (~1 μm)	35–50 proteins	Proteome	FFPE/frozen	High (tissue architecture reconstruction)	III (clinical validation stage)	Strong pathology integration; clinically adaptable	Limited multiplexing; expensive instrumentation	(34, 35)
	MIBI-TOF	Ion beam mass imaging	Subcellular	40–60 proteins	Proteome	Frozen tissue	Very high (subcellular spatial mapping)	II–III	Ultra-high spatial resolution; immune synapse analysis	Low throughput; limited availability	(33, 36)
	MACSima/CyCIF	Cyclic fluorescence imaging	Single-cell	50–100+ proteins	Proteome	FFPE	Moderate–high	II	Flexible staining; scalable imaging cycles	Photobleaching; long acquisition time	(37)
Sequencing-based spatial omics	MERFISH/seqFISH	RNA imaging (FISH-based)	Subcellular	1,000–10,000 genes	Transcriptome	Fresh frozen	Moderate (inferred networks)	II	High molecular depth; gene-level resolution	RNA–protein discordance; cost	(38)
	Xenium (10x Genomics)	Spatial transcriptomics	Single-cell	300–5,000 genes	Transcriptome	FFPE/fresh frozen	Moderate	III	Clinical pipeline integration improving	Limited protein inference	(38, 39)
	Stereo-seq	High-density spatial transcriptomics	Near single-cell	Whole transcriptome	Transcriptome	Fresh frozen	Moderate–high	II	Large-scale spatial mapping	Data complexity; resolution variability	(40)
AI-assisted virtual spatial proteomics	Histopathology AI (e.g., virtual CODEX)	Deep learning inference from H&E	Cell-level inferred	Inferred proteome	Predicted protein spatial map	FFPE	Emerging (pattern-level)	I–II	Scalable; low cost; retrospective cohort utility	Biological interpretability; validation required	(41, 42)

In recent years, the application of IMC and CODEX technology has provided convincing case evidence, proving the importance of spatial proteomics platforms in biology and clinical practice. For example, the analysis of tumor-related lymph and myellary structure based on CODEX reveals a highly ordered immune microregion, in which different T cell subgroups, B cells and medullary cell groups form spatially separated but functionally coordinated compartments, which highlights that tumor immune regulation is strongly dependent on spatial tissue rather than simple The abundance of cells (44, 50, 51). Similarly, studies based on IMC melanoma and epithelial tumors have also found discrete immunosuppressive microenvironments, which are characterized by the spatial colocation of exhausted CD8+ T cells, Tregs and immunosuppressive medullary cell groups (52, 53). These spatially defined microenvironments are related to immune escape phenotypes and are considered to be structurally correlated with therapeutic drug resistance, which directly proves that spatial structure is closely related to treatment results. In general, these studies show that CODEX and IMC are not only high-resolution mapping technologies, but also powerful discovery platforms, which greatly enhance people’s understanding of space immune systems and immunosuppressive niche biology (54).

Although these methods significantly improve scalability and reduce immunosuppressive niche experimental complexity, their robustness in different tissue types and universality in independent clinical cohorts are still under active research. Importantly, in addition to technical differences, a common challenge faced by all platforms is the lack of standardization of spatial feature definition and analysis processes. Differences in cell segmentation strategies, neighborhood radius selection and interaction scoring algorithms will lead to significant deviations in downstream biological interpretation (55–57). In addition, most existing platforms are mainly developed for research environments. Due to their high cost, long processing time and the need for special instruments, their application in clinical workflow is still limited. Therefore, although spatial proteomics technology together provides unprecedented organizational structure resolution, its transformation value depends not only on technological progress, but also on the establishment of a standardized analytical framework and clinically compatible workflows.

### 2.3 Hierarchical organization of tumor immune spatial architecture

The latest progress in spatial single-cell proteomics shows that the TIME is not a random tissue, but follows a hierarchical spatial structure characterized by multi-scale cellular tissue. In order to unify the existing terminology and analysis framework, we propose the spatial immune ecosystem hierarchy (SIEH), which divides tumor tissue into three interrelated levels: cell neighborhoods, cell communities and immunosuppressive microenvironments.

On a microscopic scale, cellular neighborhood is the most basic spatial unit, which is defined by short-range physical proximity and the probability of direct intercellular interaction. These units capture direct immune contact such as CD8+ T cell-tumor interface or macrophage-Treg cell proximity, which mainly reflects the geometric structure of local interaction (58, 59). On the middle scale, the cell community is a recurring and structured collection of multiple neighborhoods with common composition and functional characteristics. These communities represent a stable spatial motifs and code coordinated signal conduction programs, indicating that immune regulation depends not only on the interaction between individuals, but also on higher-order spatial patterns (57). On a macroscale, the immunosuppressive microenvironment represents an active ecological unit, which is characterized by coordinated multicellular interaction and continuous immunomodulation signal network. Unlike purely structural structures, these microenvironments are defined by their biological functions, including immunosuppression, metabolic remodeling and cytokine-mediated enhancements. Typical examples include macrophage-Treg co-locating microenvironment and the matrix barrier of immune rejection (60). Importantly, this hierarchical structure suggests a conceptual shift, that is, from cell or marker-centered analysis to interpreting tumor immunity from the ecosystem level, in which the spatial structure itself is the decisive factor of immune function and therapeutic response (61).

### 2.4 Spatial proteomics data analysis process

In addition to the advancement of data acquisition technology, the analysis of spatial proteomics data also relies on a series of computational processes aimed at quantifying cellular colocation, spatial interaction and higher-level organizational structure. These analytical frameworks are crucial to converting high-dimensional imaging data into biologically significant spatial features. The first type of method focuses on the quantification of cell colocation, which is used to evaluate the spatial proximity or co-presentation of specific cell groups. Common methods include distance-based measurement, spatial correlation analysis and point process statistics (such as Ripley’s K function), which can quantitatively evaluate whether specific cell types are aggregated or separated in the tissue area (58, 62, 63). The second type of method involves neighborhood enrichment analysis, which is used to assess whether certain cell types are overexpressed or underexpressed in the local microenvironment of reference cell groups. These methods usually rely on an arrangement-based statistical framework or k-neighbor graph to determine whether the observed spatial association deviates from the random spatial distribution. This method is widely used to characterize the interaction of immune cells in the tumor microenvironment (64, 65). The third level of analysis focuses on microenvironmental identification and spatial domain clustering, aiming to divide the tissue into microzones with different functions based on spatially resolved cell composition. Map-based clustering methods, including community detection algorithms applied to spatial cell maps (for example, Leiden clustering or Louvain clustering), and spatial perception probability models, have been increasingly used to define tumor microenvironment and microenvironment compartments (66, 67).

Recently, an integrated framework combining graph neural networks and machine learning methods has been born to jointly model spatial structures and molecular characteristics, so as to characterize tumor ecosystems more finely. Despite these progress, the standardization of the analysis process across studies is still a major challenge, because the differences in segmentation strategies, neighborhood definitions and clustering parameters will significantly affect the biological interpretation (68, 69).

## 3 Tumor immunosuppressive niches: spatial organization and biological significance

### 3.1 From immunosuppressive cells to immunosuppressive niches

Immunosuppression in tumors is usually attributed to the accumulation of inhibitory cell types, including Tregs, tumor-related macrophages, medullary inhibitory cells and cancer-related fibroblasts (70, 71). Although the higher density of these populations is usually associated with poor prognosis and reduced immunotherapy response, these relationships are not consistent in different tumor types or patient cohorts, suggesting that cell counts alone cannot fully explain immune dysfunction. Spatial analysis provides a complementary perspective (72, 73). Immune cells, stromal cells and malignant cells occupy a specific area in the tumor, and their local neighborhood forms biological activity. In these regions, cells communicate through receptor-ligand interactions, soluble factors, chemokine clues and metabolic interactions, thus creating a microenvironment that regulates immune function (74). Therefore, immunosuppression is produced by coordinated multicellular interaction, not the existence of a single inhibitory cell. This framework stimulates the concept of immunosuppressive ecology: a spatial tissue microenvironment that supports immune escape and tumor progression (15, 61). By emphasizing the collective behavior of interacting cell groups, the ecological position model goes beyond the traditional cell-centered explanation and emphasizes that spatial tissue is a key determinant of immune regulation (16).

### 3.2 Spatial tissue is the determining factor of local immune state

Spatial proteomics emphasizes the influence of local cell environment on tumor immune behavior (58). Many regulatory processes, including receptor-ligand interaction, cytokine signaling and competition for nutrients, work within a limited space (25). Therefore, the molecular penotype of a cell does not always predict its functional state. Cells with similar transcription or proteomic characteristics can show significantly different behaviors, depending on the microenvironment around them (75). High-dimensional imaging studies show that local cellular tissue patterns usually provide more insights than measuring single-cell abundance (76). For example, cytotoxic T cells near malignant cells are usually associated with active anti-tumor responses, while T cells embedded in inhibitory regions usually show signs of dysfunction or exhaustion (77, 78). These observations show that immune activity is formed by the collective characteristics and interactions of neighboring cells, not by individual cell groups. In order to analyze this high-level organization, spatial research has introduced frameworks such as cellular neighborhoods, cellular communities and spatial ecosystems. Although these methods vary in scale and method, they are all aimed at identifying repeated patterns of cellular colocation associated with specific biological functions (79). In short, these results emphasize that tissue structure constitutes another layer of immune regulation, which strengthens the importance of spatial tissue in understanding tumor immunity.

### 3.3 Representative immunosuppression niche revealed by spatial proteomics

Spatial proteomics studies on a variety of tumor types consistently show that tumor immune regulation is not only affected by the abundance of immune cells, but also by the spatial tissue at higher levels in their tissue structure. These findings together suggest that the functional immune state does not originate from isolated cell groups, but from a structured cell collection. In a landmark triple-negative breast cancer study, Keren et al. used MIBI technology to characterize the spatial structure of the tumor microenvironment and identify different cell communities composed of tumor cells, macrophages, lymphocytes and matrix components (43). Importantly, the study found that the prognosis of patients is not only related to the abundance of immune cells, but also to the composition and spatial arrangement of these cell communities, which initially confirms that the space tissue itself carries prognostic information and represents a fundamental layer of tumor biology. Similar observations have also been reported in colorectal cancer. Researchers such as Schürch used CODEX imaging technology to construct a high-dimensional spatial map of the TIME and identified ordered immune communities rich in macrophages, dendritic cells and T lymphocytes. These structured immune communities are associated with enhanced immune activation and good clinical prognosis, while tumors that lack this coordinated spatial tissue have a poor prognosis, highlighting the importance of spatial coordination in building effective anti-tumor immunity (44). In HCC, recent spatial proteomic analysis has further expanded these findings, confirming that orderly cell communities are closely related to postoperative recurrence and tiny residual foci (17). These spatially structured immune ecosystems are characterized by coordinated interaction between immune cells and matrix cell populations, together forming a continuous immunosuppressive microenvironment, which may support tumor survival and disease recurrence. In these tumor types, a common principle emerges: immunosuppression and immunomodulation states are not only determined by a single cell group, but are encoded by spatially orderly cellular ecosystems. This transformation from cell-centered to structure-centered explanation provides a conceptual framework for understanding the formation and persistence of immunosuppressive microenvironments in cancer (44). In order to further support the universal applicability of the spatial immunosuppressive microenvironment in different tumor types and analysis platforms, we have summarized the representative evidence from the research of breast cancer, colorectal cancer and HCC in **Table 2**.

**Table 2:** Cross-cancer evidence supporting spatial immunosuppressive niches.

Cancer type	Platform	Key niche features	Signaling axis	Functional outcome	Representative references
HCC	CODEX	VIM^+^ TAM–Treg colocalization	IL-1β	Immune suppression	(17)
PDAC	IMC/CosMx	Myeloid–Treg enrichment niche	CSF1R/IL-1	T cell dysfunction	(80)
CRC	CODEX	Immune exclusion niche	CXCL12	Poor infiltration	(44)
NSCLC	IMC	Myeloid dominant niche	IL-1/TGF-β	Exhausted CD8^+^ T cells	(81)
Breast cancer	MIBI	Structured immune neighborhoods	multiple axes	Prognosis stratification	(3)

HCC, hepatocellular carcinoma; PDAC, pancreatic ductal adenocarcinoma; CRC, colorectal cancer; NSCLC, non-small cell lung cancer.

## 4 A macrophage–Treg immunosuppressive niche revealed by spatial single-cell proteomics

### 4.1 Identification of a VIM-highly macrophage-associated suppressive niche

**Figure 1:**
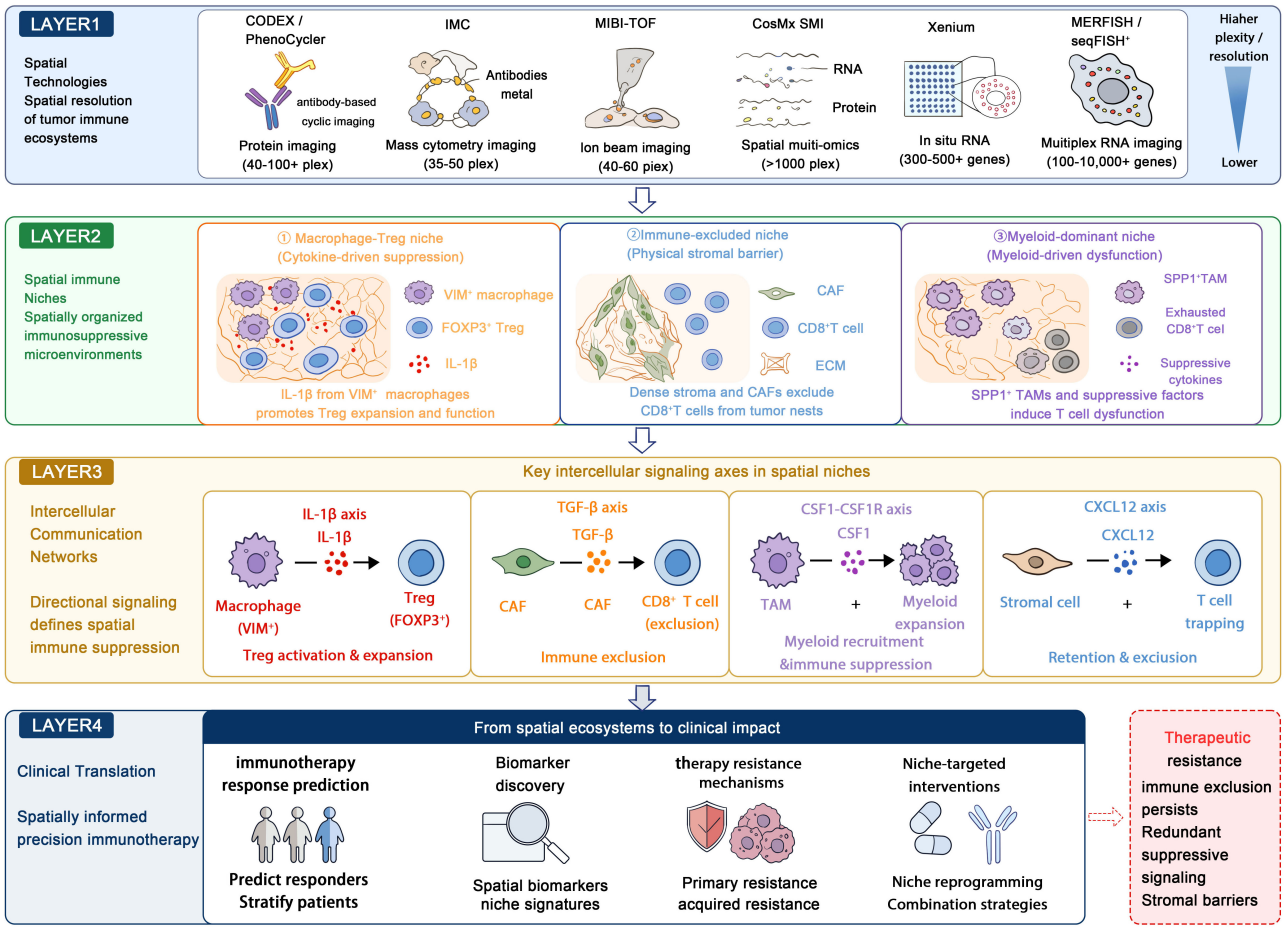
A spatial immunosuppressive ecosystem framework linking spatial proteomics technologies, tumor immune niches, and therapeutic translation Spatial technologies (including CODEX, IMC, MIBI, CosMx SMI, Xenium, and MERFISH) enable high-resolution mapping of tumor immune ecosystems and reveal distinct immunosuppressive niches, including macrophage–Treg, immune-excluded, and myeloid-dominant microenvironments. These spatially organized niches are governed by key intercellular signaling axes, including IL-1β, TGF-β, CSF1R, and CXCL12 pathways, which coordinate immune suppression, cellular exclusion, and myeloid remodeling, thereby shaping therapeutic response and resistance.

Although spatial proteomics studies have confirmed the presence of immunosuppressive niches in various tumor types, the cellular composition and molecular mechanisms of these structures remain not fully understood. Recent studies in HCC have provided important mechanistic insights by identifying a previously unrecognized macrophage-associated suppressive ecosystem. Qiu et al. used CODEX-based spatial proteomics combined with single-cell transcriptomics to characterize the spatial structure of HCC and identified a macrophage population marked by high expression of VIM (17). Compared with other subtypes of macrophages, VIM-highly expressing macrophages exhibit distinct transcriptional and functional features, including enrichment of inflammatory signaling pathways and high expression of Interleukin-1β(IL-1β) (82). These cells are unevenly distributed within the tumor and preferentially localize to areas enriched for Tregs, forming a consistent spatial pattern in patients, which is further illustrated in **Figure 1**. Notably, this spatial arrangement suggests that the biological effects of macrophages and Tregs may be interdependent, in contrast to previous approaches that viewed their infiltration independently as a prognostic indicator. In other words, immunosuppression may not originate solely from the presence of macrophages or Treg cells, but rather from the formation of a stable macrophage-Treg interaction network within a limited tissue space (83). Conceptually speaking, this discovery further supports the shift of immune regulation research from a cell-centered model to an ecosystem-centered model. At the same time, it also highlights a key advantage of spatial proteomics: it can identify functionally correlated cell combinations that cannot be inferred from cell abundance alone.

### 4.2 Enhancement of cytokine-mediated immunosuppression space microenvironment

An unresolved key problem in tumor immunology is how to maintain the spatial immunosuppressive microenvironment for a long time under the condition of continuous renewal of immune cells and therapeutic interference. Recent studies have emphasized that cytokine-mediated feedback loops are a potential mechanism for maintaining these structures. In HCC, spatial transcriptomics and functional verification studies show that IL-1β is a key mediator connecting macrophage activity and regulatory T cell (Treg) function regulation. Macrophages with inflammatory activation characteristics show increased expression of IL-1β, which is related to the enhanced inhibitory transcription program in neighboring Treg cells. Experimental evidence further shows that the IL-1 signaling pathway participates in shaping the state of local immune cells by modulating both myeloid recruitment and T cell differentiation trajectories (17). In addition to HCC, the role of IL-1 family cytokine as a regulating factor of spatial immune tissue is also increasingly valued. In pancreatic cancer and colorectal cancer, the IL-1 signaling pathway is related to the formation of immunosuppressive microregions rich in medullary cells and the exclusion of cytotoxic T cells from the tumor nest (84–86). These findings show that IL-1β may not only act as a soluble inflammatory medium, but also as a spatial organizer to stabilize the immunosuppressive microenvironment through a local feedback mechanism (87). In summary, these observations support a model that IL-1β of macrophages-derived helps to maintain a self-enhanced immunosuppression loop, in which the spatial proximity between macrophages and Tregs is both the cause and the result of persistent immune dysfunction (88).

In addition to the evidence that has been established so far, an important conceptual question is: does the IL-1β-driven spatial tissue represent the universal principle of tumor immune ecology, or is it limited to the situational dependence of specific inflammatory tumor types? (89). From the perspective of the spatial system, IL-1β may not only act as an effect cytokine, but rather as a “context-sensing regulator” that amplifies local immune imbalances once a critical density of macrophage–Treg proximity is reached. Under this framework, spatial proximity is not only the result of chemokine collection. It may actively participate in stabilizing cytokine gradients and enhancing the feedback intensity in specific tissue areas. This reminds us that the immunosuppressive microenvironment may be an emerging characteristic of a coupled space-signal system, in which the physical arrangement of cells and cytokine signaling evolve together to maintain functional stability (90). If this model is valid, then the treatment strategy for IL-1β or its downstream pathways may not only consider molecular inhibition, but also consider the destruction of the space tissue itself. In other words, effective reprogramming of the TIME may require “deconstructing the spatial feedback loop” instead of suppressing a single signal node (87, 91).

### 4.3 Clinical significance and spatial prognosis value of macrophage-Treg microenvironment

From a clinical perspective, the evidence accumulated by spatial proteomics and spatial transcriptomics research in the past five years shows that the prognostic value of tumor-infiltrating immune cells is not solely determined by their abundance, but is strongly influenced by their spatial organization within the tumor microenvironment. In HCC, the density of macrophage-Treg spatial colocation is associated with more aggressive tumor phenotypes and adverse clinical outcomes, suggesting that immunosuppression is regulated not only at the molecular level but also at the organizational structure level (17). Importantly, this spatial prognostic signal cannot be simplified into the independent contribution of macrophages or Tregs. On the contrary, recent spatial modeling studies using neighborhood enrichment analysis and intercellular proximity indicators show that the interaction structure between these two cell groups contains additional predictive information beyond the traditional immune infiltration score (76, 92, 93). This observation is consistent with other emerging findings in the field of solid tumors, that is, the spatial interaction pattern (rather than the absolute immune cell count) is more correlated to the efficacy of immune checkpoint blocking therapy. The latest advances in spatial statistics and computational pathology make it possible to characterize these microenvironments more quantitatively. Methods such as spatial self-correlation analysis (for example, Moran’s I-based method), cell interaction network based on graphs, and neighborhood density scoring systems have been used to quantify immune cell aggregation and space-dependent structures (94). These Methods Jointly Support The View That The Macrophage-Treg Microenvironment Represents Discrete Functional Space Units, Which Can Be Defined By Mathematical Methods And Repeatable Measurements On Different Imaging Platforms (95).

However, despite these progresses in methodology, clinical transformation still faces several key challenges. First of all, there is no universally accepted definition of spatial “neighborhood” at present, and parameter selection (such as radius threshold or k nearest neighbor setting) will significantly affect the subsequent biological interpretation. Secondly, most space biomarkers have been verified in retrospective cohort studies, and their predictive robustness in prospective multicenter clinical trials is still limited. Third, due to computational complexity and lack of standardized reporting framework, the integration of space biomarkers into the routine pathological workflow is still limited (61). In addition to technical considerations, an important conceptual significance is that the spatial immune microenvironment may represent a missing link in the current biomarker paradigm. Traditional biomarkers, such as PD-L1 expression, tumor mutation load or immune cell density, can only reflect molecular state or component characteristics, but cannot explain the interaction topology in the tissue. In contrast, the macrophage-Treg microenvironment reflects the higher-order organizational principle of tumor immunity, in which the functional results are determined by coordinated spatial interaction rather than isolated cell state. From this perspective, some people believe that the future prognostic model should go beyond the “cell-centered” indicator and shift to the “interaction-centered” spatial framework (96, 97). This method can not only improve the accuracy of prediction, but also characterize the immune ecology of tumors more deeply. However, transforming this concept into clinical practice requires a standardized definition of spatial characteristics, reliable cross-platform verification, and integration with the existing histopathology workflow. In a word, the macrophage-Treg space microenvironment is not only a biological tumor immunomodulation unit, but also a clinically operable unit, but its full play to its transformation potential depends on bridging the gap between space biology and clinical pathology (98).

These findings support a hierarchical spatial framework in which tumor microenvironment represents the global context, tumor ecosystems describe interaction networks, and immunosuppressive niches represent the minimal functional units of immune regulation. In short, these findings show that spatial tissue rather than mere cell abundance is the fundamental determinant of tumor immunity, thus redefining the concepts of prognosis and predictive biomarkers in cancer immunology.

## 5 From spatial niches to therapeutic intervention

### 5.1 Immunosuppressive niches as therapeutic targets

**Figure 2:**
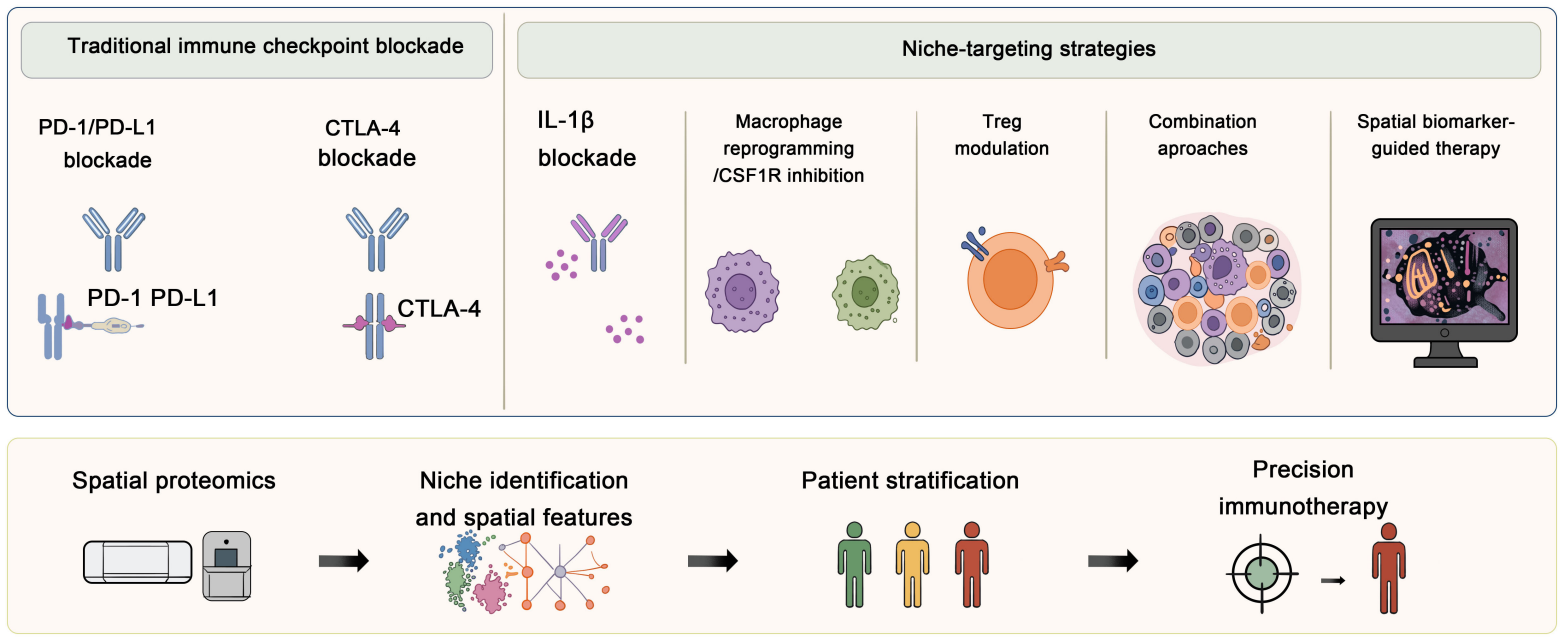
Therapeutic strategies targeting immunosuppressive niche.

Most current immunotherapies are designed to block a single signaling pathway, such as PD-1/PD-L1 or CTLA-4 (5). While these approaches can restore T cell activity in some patients, they do not directly target the spatial organization of the TME. The concept of an immunosuppressive niche introduces a different therapeutic perspective (99) (**Figure 2**). If immunosuppression is maintained by a coordinated cellular ecosystem, then disrupting the interactions that maintain these ecosystems may represent an effective therapeutic strategy. In this framework, the therapeutic target is no longer a single molecule, but a functional network of interactions. This view is supported by a growing body of evidence showing that macrophages, Tregs, fibroblasts, and other stromal cell populations work together to establish a local immune barrier (100). Targeting a component of this network may alter the stability of the entire niche and potentially improve responsiveness to immunotherapy.

### 5.2 Targeting the VIM-highly macrophage–Treg axis

The identification of IL-1β-dependent communication between VIM-highly macrophages and Tregs suggests several potential intervention strategies. One approach is to interfere with IL-1 signaling. Clinical agents targeting IL-1β have already been evaluated in inflammatory diseases and certain malignancies, providing a potential translational pathway for future investigation (101, 102). Another possibility is to modulate macrophage recruitment, survival, or polarization. Therapeutic strategies targeting CSF1R and related pathways have shown the ability to reshape myeloid cell populations in preclinical and clinical studies (103, 104). Although results have been mixed, these approaches highlight the feasibility of manipulating macrophage-dependent niches (105). Importantly, the goal of niche-targeted therapy may not be complete elimination of a particular cell population. Instead, reprogramming spatial interactions and restoring a permissive immune microenvironment may represent a more realistic therapeutic objective.

### 5.3 Future directions: from molecular biomarkers to spatial biomarkers

Perhaps the most important contribution of spatial proteomics is the introduction of spatially based biomarkers. Traditional biomarkers mainly quantify the presence or absence of specific molecules, while spatial biomarkers integrate information about cell localization, proximity relationships, and neighborhood structure (76). Recent studies have shown that measurements such as intercellular distance, neighborhood enrichment, and niche density may be related to prognosis and treatment response (106). Although these methods are currently mainly limited to the research environment, they provide a framework for integrating tissue architecture into precision oncology. We believe that the future clinical value of spatial proteomics may lie more in defining clinically actionable spatial features than in identifying more cell types. With the increasing standardization of imaging techniques and the continuous maturation of computational methods, spatial biomarkers are expected to eventually complement molecular profiling analysis and contribute to more accurate patient stratification (41).

## 6 Spatial features as a new dimension for precision immunotherapy

### 6.1 Why are new biomarkers needed?

Clinical practice of immune checkpoint blockade therapy has spurred extensive research into predictive biomarkers. Currently, PD-L1 expression levels, tumor mutational burden (TMB), microsatellite instability (MSI), and TILs remain the most widely studied indicators of immunotherapy response. However, the predictive performance of these biomarkers remains inconsistent across different tumor types and clinical settings. A significant limitation is that most biomarkers currently in use are based on molecular abundance. They quantify the presence or absence of genes, proteins, or cell populations, but provide little information about tissue spatial structure (72, 107). Therefore, similar levels of PD-L1 expression or immune-cell infiltration did not necessarily translate into similar clinical outcomes. Spatial proteomics studies have shown that tissue structure provides biological information that traditional biomarkers cannot (97). In multiple tumor types, the spatial distribution of immune cells has a stronger association with patient prognosis and treatment response than immune cell abundance alone. These results emphasize that immune activity is not only composed of cells but also determined by spatial relationships between cells and interactions occurring in the local tissue environment (99). In this context, immunosuppressive niches can be viewed as functional units of tumor tissue, where clinically relevant information is embedded in spatial interaction networks rather than individual molecular markers.

### 6.2 Construction and interpretation of spatial biomarkers

Spatial single-cell proteomics enables the quantitative study of tissue architecture, thus creating an opportunity to obtain biomarkers that capture the tissue characteristics of the TME. Several analytical strategies have emerged to describe spatial relationships between cells and quantify niche structure. One commonly used approach focuses on spatial proximity. Indicators such as the distance between macrophages and Tregs, the radius of rejection of CD8^+^T cells or the proximity of immune cells to tumor cells (typically interpreted within short-range interaction thresholds, e.g., <20 μm in high-resolution spatial profiling studies) provide information about the local interaction potential (58). Because many regulatory processes depend on short-range communication, these measurements may provide a better reflection of functional relationships than cellular abundance alone. The second set of biomarkers is based on cellular neighborhoods (108). Neighborhood enrichment scores assess the tendency of specific cell populations to occupy a shared space using permutation-based or normalized statistical frameworks (e.g., z-score or empirical p-value thresholds) and can reveal recurrent patterns of cell organization. In breast and colorectal cancer, neighborhood components are associated with patient survival and immune activity, suggesting that the local cellular environment carries biologically and clinically relevant information (37).

There is also growing interest in ecosystem-level analyses that capture higher-order spatial organization (109). Rather than studying isolated pairs of cells, these frameworks model coordinated interactions between multiple cell populations and signaling programs (110, 111). These strategies are particularly relevant to immunosuppressive ecology, because macrophages, Tregs, fibroblasts, endothelial cells and malignant tumor cells jointly shape the local immune state. The resulting spatial ecosystem can reflect the biological characteristics of tumors more comprehensively than a single interaction indicator, particularly when consistent analytical definitions are applied across studies. One of the main obstacles to clinical transformation is the lack of methodological standardization, particularly in the definition of cell neighborhoods, interaction distance thresholds, and computational processes in different studies, which limits repeatability and cross-cohort comparison (112). Establishing a consensus analysis process and robust spatial metrics is crucial for integrating spatial biomarkers into future clinical practice (69).

### 6.3 Clinical opportunities and translational barriers of spatial proteomics

Space biomarkers have attracted much attention because they can capture organizational-level structural information that traditional molecular measurements cannot reflect (29, 55). In tumor tissue, the spatial proximity and interaction between macrophages, Tregs, tumor cells and matrix components form an additional bioregulatory layer, which may affect the therapeutic response. For example, tumors rich in macrophage-Treg-related immunosuppressive spatial structures often show different sensitivity to interventions targeting IL-1 signaling pathways, macrophage plasticity or regulatory T cell function, which indicates that spatial tissue may provide supplementary information for risk stratification and treatment options (41, 113). However, despite these encouraging findings, the transformation of spatial proteomics into conventional clinical pathology remains a challenge (95, 114). A major limiting factor is the inherent high cost and technical complexity of the current spatial analysis platforms, which require dedicated instruments, rich experimental experience and computationally intensive analysis processes. These requirements are difficult to reconcile with the standardized, high-throughflow and time-sensitive workflow of clinical diagnosis and pathology. In addition, the differences in the analysis process and the lack of a universal standardized definition of spatial characteristics hinder the repeatability between different studies and patient cohorts. Therefore, many reported spatial biomarkers still rely mainly on retrospective analysis and lack evidence of prospective clinical verification (29, 41). In parallel, emerging engineering-based approaches, such as microrobot-assisted drug delivery systems, represent complementary efforts to improve the precision and controllability of tumor-targeted interventions, although they remain largely independent of spatial omics-based biomarker frameworks (115). As a result, they can only be used for exploratory or prognostic evaluation at present, and cannot be used as operational clinical decision-making tools (116).

In summary, these challenges show that the key obstacles to clinical application lie not only in technical limitations, but also in the lack of a robust framework that can transform high-dimensional spatial measurements into stable, repeatable and clinically significant characterization. Therefore, future progress will depend on establishing standardized analytical strategies, improving cross-platform repeatability, and generating forward-looking evidence to prove its clinical application value beyond existing pathological and molecular parameters (Neuronal Reshaping of the Tumor Microenvironment in Tumorigenesis and Metastasis: Bench to Clinic) (117).

In the end, the transformation value of spatial proteomics will not depend on the resolution or complexity of spatial data collection, but on whether these high-cost and analytical heterogeneity measurements can be refined into robust, standardized and clinically explainable spatial characteristics, thus compatible with conventional pathological decision-making.

## 7 Conclusions and future perspectives

Spatial single-cell proteomics has greatly changed our understanding of tumor biology by realizing the *in-situ* characterization of cell composition, spatial tissue and intercellular communication within the complete tissue structure. In addition to its transformation applications, such as biomarker development, patient stratification and therapeutic target identification, the more fundamental contribution of spatial analysis is to reveal the organizational principle of regulating tumor-immune interaction. A key emerging biological insight is that tumor behavior is not solely determined by the abundance of specific cell populations, but is critically shaped by localized cell–cell crosstalk within spatially constrained microdomains. These interactions form a functional immunosuppressive microenvironment, which actively regulates the state of immune cells and may lead to therapeutic drug resistance. Against this background, drug resistance should not only be regarded as the result of internal changes in tumor cells, but also as the emergence characteristics of the spatial tissue cell network that maintains inhibitory signaling pathways. In addition, the spatial heterogeneity of immune cell distribution represents the basic attributes of tumor ecosystems, rather than mere descriptive biomarker characteristics. Distinct spatial immune architectures correspond to functionally divergent microenvironments, which may underlie the variability in treatment response observed across patients. Therefore, spatial characteristics can not only provide predictive clinical information, but also reveal the deeper biological principles of regulating tumor tissue. Despite these advances, there are still some challenges before spatial analysis is fully integrated into routine clinical practice. These challenges include a lack of standardized definitions of spatial characteristics, differences in computational processes, and limited forward-looking verification of multicenter queues. Addressing these limitations is crucial to transform space biology into a clinically operable tool.

Looking forward, the most impactful direction may lie in integrating spatial, molecular, and functional data to construct a unified framework of tumor ecosystems, in which cellular interactions, spatial organization, and signaling dynamics are jointly modeled. Against this background, emerging artificial intelligence methods, including graph neural networks and Transformer-based architectures, may be able to learn spatial interaction networks directly from tissue slices, so as to realize automatic immune microenvironment segmentation and accelerate the clinical interpretation of space biomarkers. This integrative paradigm may eventually shift cancer research from a cell-centered paradigm to an interaction-centered paradigm, in which the treatment strategy aims not only to target a single molecular pathway, but also to destroy the spatial structure that maintains tumor immune dysfunction.
